# Inverted multi-layer internal limiting membrane flap for macular hole retinal detachment in high myopia

**DOI:** 10.1038/s41598-022-14716-7

**Published:** 2022-06-22

**Authors:** Xianggui Wang, Ying Zhu, Huizhuo Xu

**Affiliations:** 1 Hunan Key Laboratory of Ophthalmology, Eye Center of Xiangya Hospital, Central South University, Changsha, China; 2 National Clinical Research Center for Geriatric Disorders, Xiangya Hospital, Central South University, Changsha, China

**Keywords:** Retinal diseases, Surgery

## Abstract

To investigate the surgical outcomes of pars plana vitrectomy (PPV) combined with inverted multi-layer internal limiting membrane (ILM) flap for the treatment of macular hole retinal detachment in high myopia. We retrospectively analysed the medical records of macular hole retinal detachment (MHRD) patients with high myopia. The patients were divided into two groups with different surgical procedure: inverted multi-layer ILM flap group (group 1, 27 eyes) and the ILM peeling group (group 2, 29 eyes). Retinal reattachment rate, macular hole closure rate at last follow-up and BCVA at 6 months post-operation were compared between the two groups. After primary PPV and silicone oil removal, the retinal reattachment rate was 96.3% in group 1 and 93.1% in group 2 respectively at last follow-up, showing no statistically significant difference (odds ratio = 0.525, *P* = 1.000). All eyes in group 1 had type I macular closure (100%, 27/27), while only 7 eyes (24.1%, 7/29) in group 2 have type I macular hole closure. The difference was statistically significant (odds ratio = 0, *P* < 0.05). The mean logMAR BCVA both improved significantly at 6 months post-operation compared with pre-operation (t = 4.181, *P* < 0.001; t = 3.217, *P* < 0.001), however the difference of post-operation BCVA between the two groups was not statistically significant (t = 0.906, *P* > 0.05). PPV combined with inverted multi-layer ILM flap could achieve better anatomical outcomes than ILM peeling technique with no significant advantage in functional outcomes.

## Introduction

Macular hole retinal detachment (MHRD) is one of the most severe complications in high myopia and severely impairs the visual function of patients^1,2^. The conventional surgical approach is pars plana vitrectomy (PPV) combined with ILM peeling and gas or silicone oil tamponade. Complete posterior vitreous cortex (PVC) removal and ILM peeling are key factors to the success of PPV^3^. However, the closure rate of macular holes (MHs) is low^4^. Unclosed MHs may affect postoperative vision recovery and lead to recurrence of retinal detachment^5,6^. In recent years, the application of ILM flap inversion or insertion technique has greatly improved the MH closure rate and the success rate of retinal reattachment^2,7,8^. However, retinal pigment epithelium cells at the base of the MH may be damaged during ILM flap insertion, and at the same time, the inserted ILM affects the repair of the ellipsoid zone and the external limiting membrane (ELM) at the MH, which, in turn, affects patient visual acuity^9^. Therefore, most experts prefer the inverted ILM flap technique. However, the single-layer inverted ILM flap is prone to displacement or reverse inversion, leading to surgical failure. Thus, we focused on improving the inverted ILM flap technique in our study. We performed a retrospective analysis of 23 gauge(23G) PPV combined with the inverted multi-layer ILM technique treating MHRD in high myopia, with PPV in combination with ILM peeling as the control group. The differences in best-corrected visual acuity (BCVA), MH closure rate, and the success rate of retinal reattachment were compared between the two groups to evaluate the efficacy of the multi-layer inverted ILM technique for the treatment of MHRD in high myopia.

## Patients and methods

### Patients

The protocol for this retrospective study was approved by the Ethics Committee of Xiangya Hospital, Central South University, Hunan, China. The study adhered to the tenets of the Declaration of Helsinki, and all the enrolled patients obtained informed consent and signed informed consent forms before participation.

In this retrospective study, We reviewed the medical records of all patients with highly myopic MHRD admitted to the Ophthalmology Department of Xiangya Hospital of Central South University from January 2018 to December 2019. The inclusion criteria were: (1) axial length (AL) ≥ 26.5 mm, (2) spherical equivalent refraction (SER) ≤  − 6.00 diopters, (3) MHRD confirmed by optical coherence tomography (OCT) examination or intraoperatively, and (4) proliferative vitreoretinopathy (PVR) < grade C. The exclusion criteria were: (1) combined peripheral retinal hole in addition to the MH, (2) previous history of intraocular surgery, (3) combined with other ocular diseases such as glaucoma, optical neuropathy or ocular trauma, (5) combined significant cataract or keratopathy affecting PPV. Routine eye examinations including best-corrected visual acuity (BCVA) measurement, intraocular pressure (IOP) measurement by Goldmann applanation tonometry, slit lamp examination and biocular indirect ophthalmoscopy, color fundus photography, A/B-mode ultrasonography, ultrasound biomicroscopy (UBM) and spectral-domain optical coherence tomography (SD-OCT, Carl Zeiss), were performed before and after surgery. BCVA was evaluated using the Snellen logarithmic standard visual acuity chart and was converted to logMAR visual acuity at the time of recording.

The patients were divided into two groups with different surgical procedure: inverted multi-layer ILM flap group (group 1) and the ILM peeling group (group 2). The retinal reattachment rate, macular hole closure rate and BCVA were compared between the two groups before and after surgery. The criteria for MH closure were established based on spectral domain-OCT (SD-OCT) examination^10,11^. Type I closure was defined as the connection and attachment of the two ends of MH to the RPE layer, While type II MH closure was defined as the attachment of the two ends of the MH to the RPE layer, with localized retinal neuroepithelial defects in the fovea and exposed retinal pigment epithelial layer. If the two ends of MH were unconnected and still elevated and cocked and SRF was present, the MH was considered unclosed.

### Surgical techniques

All patients underwent standard three-port transconjunctival 23G PPV(Constellation, Alcon). The procedures were performed by a single highly experienced ophthalmologist. For cases with combined choroidal detachment, sclera incisions were performed at corresponding positions to drain the fluid from the suprachoroidal space. In both groups, triamcinolone acetonide (4 mg/1 mL) was used to facilitate PVC removal and ERM peeling. Indocyanine green (0.125% ICG) staining was used to assist ILM peeling to the arcade. The subretinal fluid (SRF) was drained through the MH.

In group 2, the ILM was completed peeled to the arcade and removed. In group 1, the ILM at the fovea with a diameter of approximately 2 PD was preserved and the rest of the ILM was peeled to arcades, as the same range as in group 2. The reserved portion of the ILM was detached from the retinal surface but anchored at the margin of the MH to prevent ILM loss during gas-fluid exchange. The retina was reattached after thorough gas-fluid exchange. Perfluorocarbon liquid (PFCL) was injected and the partially peeled ILM was further peeled to the MH margin. Under PFCL, a 360 degree perifoveal ILM flap were flipped from superior, temporal, nasal and inferior to form a multi-layer flap covering the MH (Surgical video as supplementary material 1). No part of the ILM was inserted below the minimum aperture of the macular hole.

Retinal photocoagulation was performed in eyes with peripheral degeneration. Gas-fluid exchange and silicon oil tamponade were performed in all eyes. The patients were asked to maintain a prone position for 6–8 h daily for 2 weeks. The silicone oil removal was performed 1–3 months after PPV according to IOP control and fundus status. In cases with combined cataract, phaco-emulsification and intraocular lens (IOL) implantation were performed at the time of silicon oil removal.

### Statistical analyses

Statistical analysis was performed using SPSS 18.0 software., and measurement data are expressed as means ± standard deviation. Age, refractive error, AL, duration of decreased vision, preoperative BCVA, follow-up period, postoperative BCVA and type I MH closure rate were compared between the two groups by independent samples t- or chi-square tests. Type I MH closure and success rates of retinal reattachment were compared by Fisher's exact tests. *P* < 0.05 was considered statistically significant.

## Results

### Demographics

Fifty-six eyes of 56 consecutive patients with highly myopic MHRD were included. Among them, 11 patients (11 eyes) were male and 45 patients (45 eyes) were female, aged 33–70 years, with a mean age of (52.71 ± 8.75) years. Among the 56 eyes, 12 eyes had posterior pole retinal detachment; 21 eyes had posterior pole retinal detachment plus peripheral detachment in 1–3 quadrants; 23 eyes had total retinal detachment, including 19 eyes with combined choroidal or ciliary body detachment. The affected eyes were divided into inverted multi-layer ILM flap group (group 1, 27 eyes) and the ILM peeling group (group 2, 29 eyes) according to the operation procedures performed during PPV. The two groups showed no statistically significant differences (*P* > 0.05) in age, refractive error, axial length, duration of decreased vision, or combined choroidal detachment ratio (Table 1).

**Table 1:** Comparison of Baseline Clinical Factors.

Charactersitic, N(%) or Mean ± SD	Inverted ILM flap group (n = 27)	ILM peeling group (n = 29)	*P*
Age, years	52.15 ± 9.56	53.24 ± 8.05	0.647
AL, mm	29.57 ± 2.35	29.28 ± 2.21	0.634
Duration of decreased vision, months	3.62 ± 5.21	4.15 ± 6.14	0.733
SEP, diopter	16.57 ± 6.70	16.12 ± 6.62	0.800
Combined CD	10 (37)	11 (38)	1

*ILM* Internal limiting membrane, *AL* Axial length, *SEP* Spherical equivalent power, *CD* choriodal detachment.

The follow-up period after surgery ranged from 6 to 12 months, with an overall mean of 8.75 months. The mean follow-up periods were (8.63 ± 2.47) and (8.86 ± 1.92) months in group 1 and group 2, respectively, showing no statistically significant difference (t = 0.3913, *P* = 0.6973).

### Comparison of anatomical and functional outcomes

At the final follow-up, the retinal reattachment rates were 96.3% and 93.1% in group 1 and group 2, respectively, with no statistically significant difference between the two groups (odds ratio = 0.525, *P* = 1.000).

**Figure 1:**
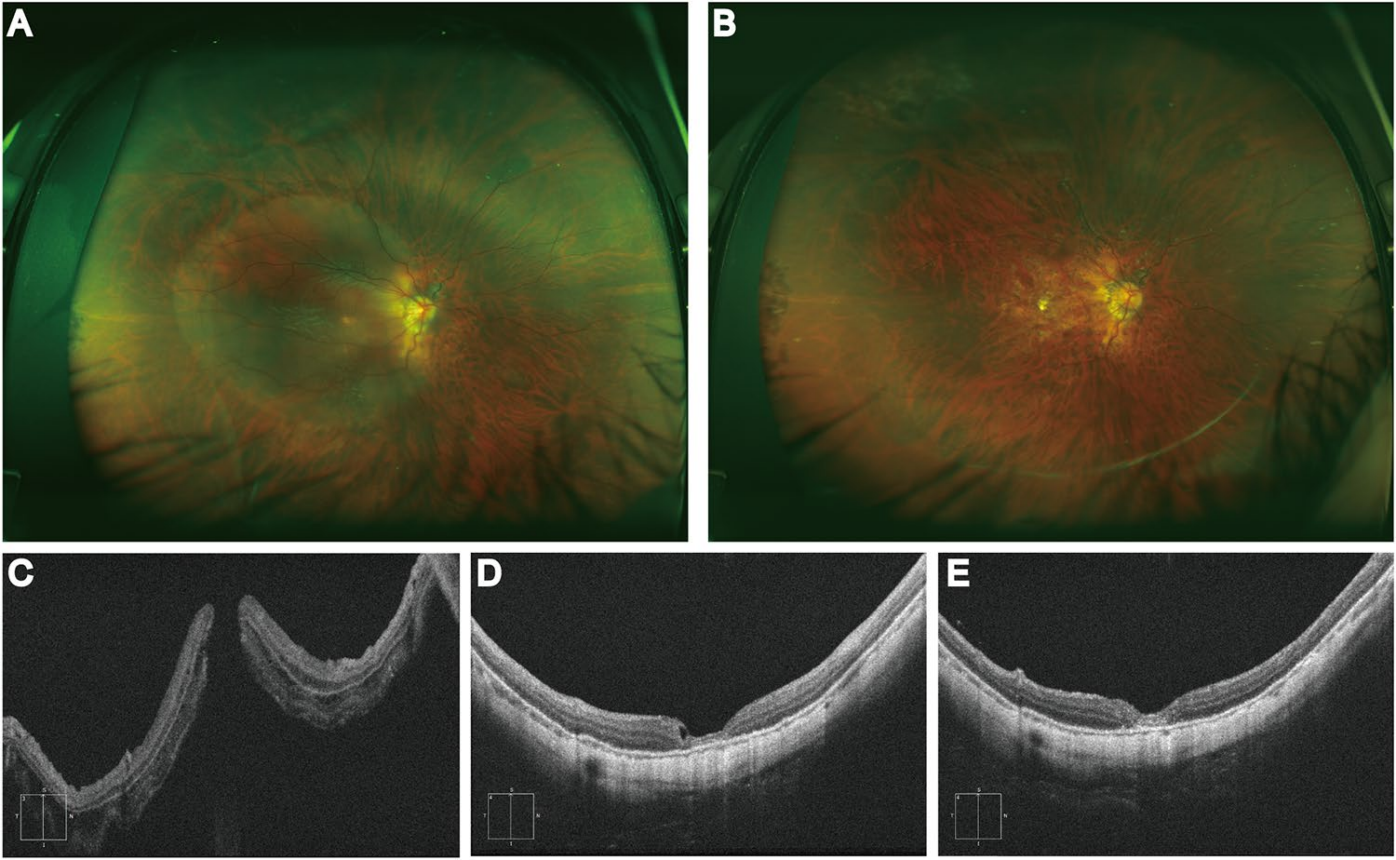
Representative images of inverted multi-layer ILM flap treating MHRD without choroidal detachment. Fundus color photos (**A**–**B**) and OCT images(**C**–**E**) of a patient in inverted multi-layer ILM flap group before and after operation. MHRD in the right eye (**A**) and retinal reattachment at 3 months after operation (**B**). OCT images showed macular retinal detachment and full-thickness macular hole before surgery (**C**). Type 1 Macular Hole closure was achieved at one months post-op (**D**) and no proliferation was observed at 3 months post-op (**E**).

**Figure 2:**
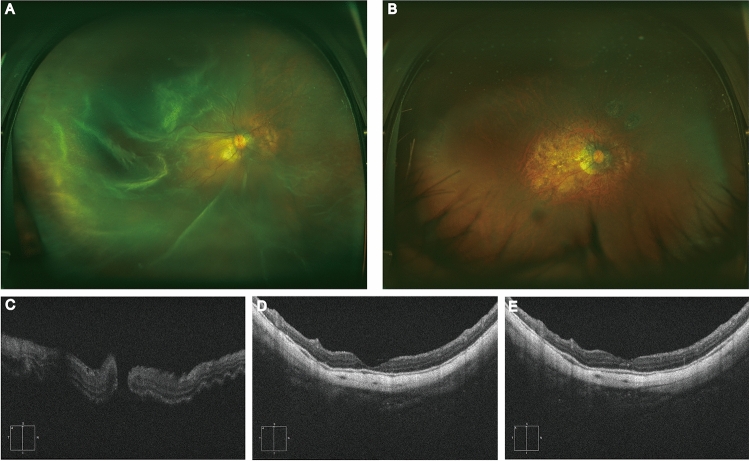
Representative images of inverted multi-layer ILM flap treating MHRD with choroidal detachment.Fundus color photos (**A**–**B**) and OCT images(**C**–**E**) of this study in right eye of a patient in multi-layer ILM inversion group before and after operation. Before operation, fundus color photos show full-thickness macular hole retinal detachment with temporal peripheral choroidal detachment of the right eye (**A**), and the retina was reattached and the macular hole healed 3 months after operation (**B**); OCT images show retinal detachment in macular area and full-thickness macular hole shown by OCT before operation (**C**), and the macular hole was closed with no proliferation and the retina was reattached at one month (**D**) and three month (**E**) after operation.

All 27 eyes in group 1 had type I MH closure (100%, 27/27) (Figs. 1, 2). But among the 29 eyes in group 2, 7 (24.1%, 7/29) had type I MH closure, 20 (69.0%, 20/29) had type II MH closure, and 2 (6.9%, 2/29) failed to close. Type I MH closure rates differed significantly between the two groups (odds ratio = 0, *P* < 0.05).

The mean preoperative logMAR BCVA values were 1.95 ± 0.76 and 1.84 ± 0.68 in group 1 and group 2, respectively, with no statistically significant difference (t = -0.551, P = 0.584). At the 6-month postoperative follow-up, the logMAR BCVA values were 1.19 ± 0.57 and 1.32 ± 0.55 in group 1 and group 2, respectively, with a statistically significant difference (t = 3.217, *P* = 0.002; t = 4.181, *P* < 0.001) compared to the preoperative BCVA of the affected eyes in both groups. However, the difference in the degree of improvement after surgery between the two groups was not statistically significant (t = 0.9799, *P* < 0.0846) (Table 2).

**Table 2:** Comparison of Surgical Outcomes. ILM internal limiting membrane, MH, macular hole, BCVA best-corrected visual acuity, logMAR logarithm of minimum angle of resolution.

Charactersitic, N(%) or Mean ± SD	Inverted ILM flap group (n = 27)	ILM peeling group (n = 29)	*P*
Follow-up duration, months	8.63 ± 2.47	8.86 ± 1.92	0.697
Type I MH closure	27 (100)	7 (24)	< 0.05*
Retinal reattachment	26 (96)	27 (93)	1.000
Preoperative BCVA, logMAR	1.95 ± 0.76	1.84 ± 0.68	0.584
Postoperative BCVA, logMAR at 6 months	1.19 ± 0.57	1.32 ± 0.55	0.369

*Fisher’s exact probability test.

### Complications

**Figure 3:**
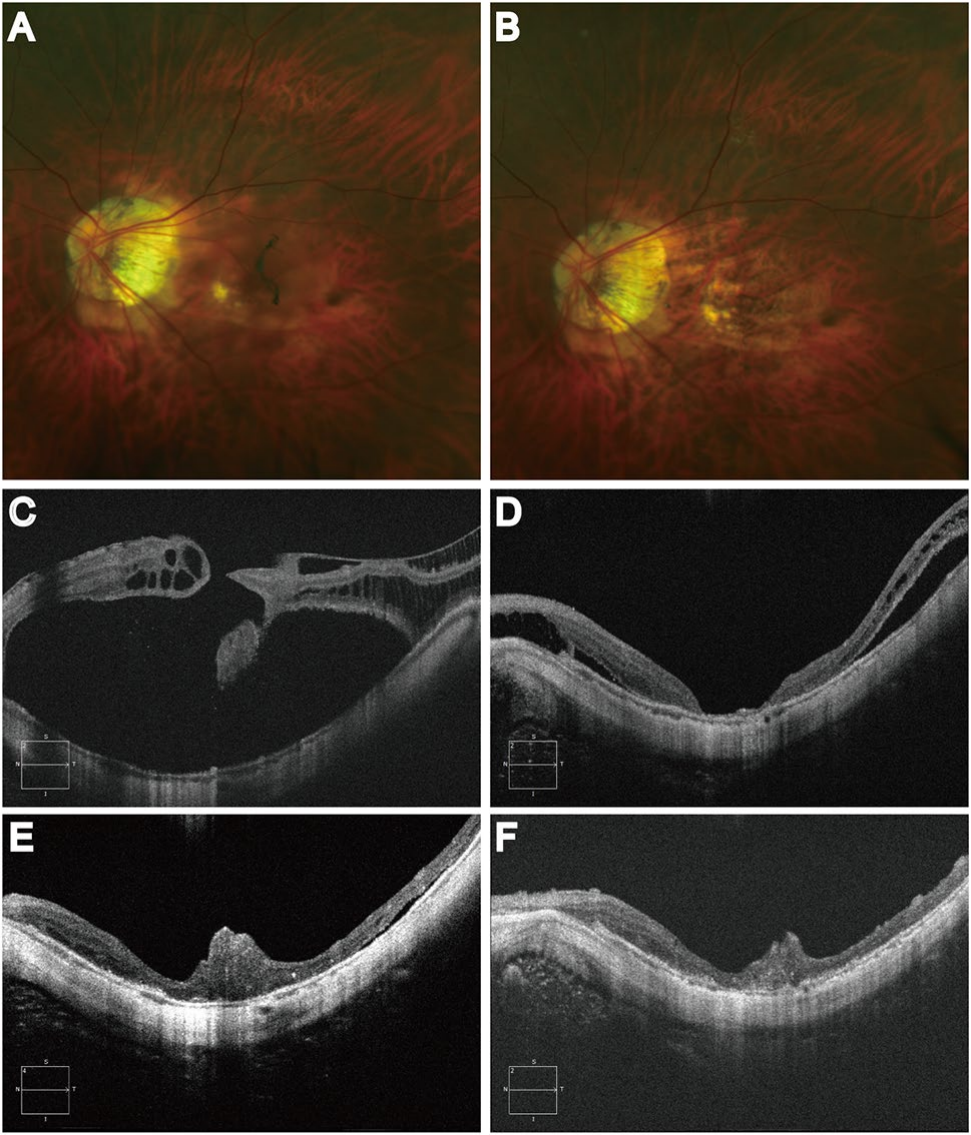
Representative images of persistent submacular fluid and post-operative glial hyper-proliferation. Fundus color photos (**A**–**B**) and OCT images (**C**–**F**) of a patient in inverted multi-layer ILM flap group before and after operation. MHRD in the left eye (**A**) and retinal reattachment at 3 months after operation (**B**); OCT images showed macular retinal detachment, full-thickness macular hole and macular retinoschisis before surgery (**C**). Type 1 Macular hole closure was achieved at 1 month post-op and no proliferation was observed (**D**). Persistent submacular fluid absorbed gradully during post-op follow-up (**D**, **E**, **F**). Glial hyper-proliferation appeared at 3 months post-op (**E**) and remained stable at 6 months post-op (**F**).

Persistent submacular fluid was observed in 11.1% (3/27) and 10.3% (3/29) of eyes in group 1 and group 2, respectively, while none reached the arcade. OCT examination showed no new-onset retinal breaks or MH reopening, and all patients had complete absorption at 3–8 months after surgery (Fig. 3). Localized perifoveal glial hyperproliferation during MH healing occurred in 25.9% (7/27) of the eyes in group 1 (Fig. 3), including 2/10 (20%) in those with choroidal detachment and 5/17 (29.4%) in those without. PVR and newly observed retinal tears after the surgery, leading to failure of retinal reattachment occurred in one case (1 eye, 1/27) and two cases (2 eyes, 2/29) in group 1 and group 2, respectively, all of whom had combined severe choroidal detachment.

In group 1 and group 2, 25.9% (7/27) and 27.6% (8/29) of patients developed high IOP after surgery, respectively, although the difference between the two groups was not statistically significant. All patients’ IOP returned to normal after the discontinuation of steroid eye drops, use of IOP-lowering eye drops, or removal of silicone oil. No serious postoperative complications, such as purulent endophthalmitis and expulsive suprachoroidal hemorrhage, were observed in any patient.

## Discussion

Vitreous surgery combined with ILM peeling is the main treatment for MHRD in highly myopic eyes^3^. However, due to the misalignment of the retina and the choroidal-scleral complex caused by the extra-long axial length and posterior scleral staphyloma, and the marked retinal and choroidal atrophy in the macular region, the surgical results are not ideal. The MH closure rate is low and retinal detachment recurs in some patients, which greatly affecting the recovery of visual acuity^12^. The combination of vitreous surgery with macular buckling or ILM inversion/insertion can greatly improve the rates of retinal reattachment and MH closure^2,7,8,13,14^. However, macular buckling is complicated and may cause complications^15^. Moreover, the required materials are not commercially available in China. Some studies^16,17^ used double-layer or free multi-layer ILM insertion by first inserting the perifoveal single-layer ILM, followed by the insertion of the free multi-layer ILM flap. But ILM insertion may damage retinal pigment epithelium beneath MH and the inserted ILM may disrupt the photoreceptor recovery, which could interfere with patient’s visual acuity recovery^18^. More recently, the commonly used inverted ILM flap technique, which uses the ILM flap to provide a scaffold for the proliferation of glial cells to repair the MH has resulted in improved MH closure rates and favorable visual outcome^19,20^. However, the removal of PVC and ERM can cause local ILM defects within the macular area, which affect ILM flap formation. In addition, the ILM flap is easily lost during intraoperative internal drainage of SRF in the MH. Moreover, the single-layer ILM flap is easily displaced or inverted reversely, leading to surgical failure. The present study utilized an improved inverted multi-layer ILM flap technique with relatively simple surgical procedures. With multiple-layer ILM flap, local defects in the flap were more tolerable than single-layer flap and it had a better coverage of over-sized MH. Also, the ILM was not peeled to the MH margin before gas-fluid exchange to minimize the risk of flap loss during the process. In this study, OCT on day 1 post-op in the inverted multi-layer ILM group showed a 100% healing rate of MH type I closure. Over time, 100% type I MH closure was observed at postoperative months 1, 3, and 6, exhibiting statistically significant differences compared to the ILM peeling group.

The primary success rate of retinal detachment surgery in the inverted multi-layer ILM group reached 96.3%, compared to 93.1% in the ILM peeling group, a difference that was not statistically significant and consistent with previous reports^8^ and probably due to the small sample size in this study. However, group 1 had a statistically significant higher MH closure rate than group 2, which could improve the retinal reattachment success rate and reduce the recurrence rate of postoperative retinal detachment. In our study, both groups showed significantly improved postoperative visual acuity. Although the postoperative visual acuity changes did not differ significantly between the two groups, some eyes in group 1 had favorable recovery of ELM and ellipsoid zone on OCT, which might be reflected on long-term visual outcomes.

Local glial hyperproliferation in the macula was also observed in 25.9% (7/27) of group 1 in this study. Although OCT of these patients showed a perfect MH healing pattern on postoperative day 1, local glial cell or Müller cell proliferation at the MH gradually appeared postoperatively due to the scaffolding effect of the ILM with the healing MH presenting a localized towering protrusion at 1 month post-op OCT and stabilized at 3 months. During follow-up, no vertical or tangential traction on the retina or macula was observed, with no cases of MH reopening or recurrence of retinal detachment. Except for the two cases with failed retinal reattachment, no PVR or ERM was observed. Previous studies showed that glial cell proliferation at the MH, which in turn affects the repair of outer retinal structures such as the ELM and the ellipsoid zone^21^, is more likely to occur after ILM insertion than after ILM inversion. In this study, 25.9% of patients in the inverted multi-layer ILM group showed glial over-proliferation at the MH for unclear reason. We suppose multiple-layer ILM flap in a relatively small MH might be one of the reason for the hyperproliferation. Thus, the inverted multi-layer ILM flap may be more beneficial to patients with large or oversized MHs. Further studies are needed to analyze the potential association of the occurrence of postoperative glial over-proliferation with MH size based on intraoperative OCT measurements of MH size after retinal reattachment.

Persistent submacular fluid was observed in 10.3% (3/29) and 11.1% (3/27) of the ILM peeling and inverted multi-layer ILM groups, respectively. No new-onset tears were found by OCT examination, with complete absorption in all patients at 3–8 months postoperatively. The literature indicated that persistent submacular fluid is more likely to occur after scleral buckling surgery of rhegmatogenous retinal detachment (RRD) than after PPV. This condition can last up to 18 months postoperatively, with no significant effect on the final degree of visual recovery after absorption^22^. The incidence of persistent submacular fluid after PPV in RRD patients varies from 0 to 15%; while its occurrence is associated with the preoperative macular detachment status and the exact mechanism remains unclear^23,24^. Previous studies have speculated that residual hyaluronic acid, protein components, lipids, saccharides and cells might cause high osmotic pressure, which results in the prolonged presence of fluid under the neuroepithelial layer in the macula^25–27^.

This study has several limitations: (1) this study was a retrospective case analysis; (2) the observation indexes were relatively simple, with only the BCVA, MH closure rate, and retinal reattachment success rate of patients observed, and indexes reflecting retinal functions such as the microperimetry and multifocal ERG were not assessed. (3) Intraoperative OCT measurements of MH size was not performed due to lack of equipment, so the size of MH was not compared between the two groups.

## Conclusion

In conclusion, 23G PPV combined with inverted multi-layer ILM flap could achieve better anatomical outcomes than ILM peeling technique with no significant advantage in functional outcomes.

## Supplementary Information

Supplementary Video 1.

Supplementary Information 1.

## Data Availability

The data used to support the findings of this study are available from the corresponding author/s upon request.
