# Supplementary material for: Inverted multi-layer internal limiting membrane flap for macular hole retinal detachment in high myopia
Source: Sci Rep. 2022 Jun 22;12:10593. doi: 10.1038/s41598-022-14716-7 (PMC9217943; doi:10.1038/s41598-022-14716-7)
Supplement: Supplementary file 2 — Supplementary Information 1. [file 41598_2022_14716_MOESM2_ESM.docx]

The surgical video of inverted multi-layer ILM flap for MHRD

Indocyanine green (0.125% ICG) staining was used to assist ILM peeling. The ILM at the fovea with a diameter of approximately 2 PD was preserved and the rest of the ILM was peeled to arcades. The reserved portion of the ILM was detached from the retinal surface but anchored at the margin of the MH to prevent ILM loss during gas-fluid exchange. The subretinal fluid (SRF) was drained through the MH. The retina was reattached after thorough gas-fluid exchange. Perfluorocarbon liquid (PFCL) was injected and the partially peeled ILM was further peeled to the MH margin. Under PFCL, a 360 degree perifoveal ILM flap were flipped from superior, temporal, nasal and inferior to form a multi-layer flap covering the MH. No part of the ILM was inserted below the minimum aperture of the macular hole.
